# Post-Herpetic Brachial Plexopathy: A Rare Case Report With a Side Note on Localizing Brachial Plexopathies and a Literature Review of Post-Herpetic Segmental Paresis

**DOI:** 10.7759/cureus.10747

**Published:** 2020-10-01

**Authors:** Hassan Kesserwani

**Affiliations:** 1 Neurology, Flowers Medical Group, Dothan, USA

**Keywords:** brachial plexopathy, painful neuropathy

## Abstract

We present the case of a 77-year-old man who developed shingles over the cervical C8 dermatome followed by post-herpetic medial cord brachial plexopathy, with hand weakness and difficulty performing the pinch " O " sign. This is the very first case, to our knowledge, of a detailed presentation of a medial cord plexopathy following shingles. We review the literature of post-herpetic brachial plexopathies and discuss the magnetic resonance imaging (MRI) findings of the brachial plexus in this group of patients. We also speculate on the intriguing finding that despite frequent abnormalities on MRI such as T2 signal hyperintensity and nerve hypertrophy, contrast enhancement of nerves is exceedingly rare. Furthermore, we adumbrate on the localization of brachial plexus lesions by proposing a user-friendly diagram and table, which simplifies the diagnostic algorithm.

## Introduction

Herpes zoster-associated brachial plexitis has also been referred to as zoster segmental paresis of the limbs. It is invariably characterized by the breakout of a shingles rash over a specific upper extremity dermatome followed by the focal weakness of an extremity.

A retrospective study by Liu et al. identified brachial plexitis in eight out of 1393 patients with herpes zoster infection. The severity of weakness varied from mild to severe. All cases were electrophysiologically characterized as axonopathies; as radiculopathy in two out of eight patients, plexopathy in two out of eight patients, radiculo-plexopathy in three out of eight patients, and mixed radiculopathy and mononeuropathy in one of eight patients. Two of the cases involved the lower limbs. A magnetic resonance imaging (MRI) study performed in half the patients early during the disease process showed nerve hypertrophy and T2 signal hyperintensity in the afflicted peripheral nerves in two patients and T2 signal hyperintensity in the dorsal horns of the cervical C5 and C6 spinal cord [1]. We will address the radiological findings later during the discussion section. Based on these numbers, the incidence of segmental weakness following shingles is quite rare.

The involvement of the cervical C8 dermatome has been described once before. This case also followed a rash. An electrophysiology study showed reduced median and ulnar motor amplitudes and denervation of C8 innervated muscles [2]. 

The varicella-zoster virus (VZV) is a herpes virus that is highly human-specific, causing varicella and zoster. In animal models, VZV-infected T cells transport the virus through the circulation, migrate into the skin, initiate blister formation, and deliver the virus to the dorsal root ganglia (DRG), where they remain latent. This is achieved by the VZV reprogramming the cell signaling pathways and disrupting the innate antiviral defenses. Furthermore, the VZV is the only human herpesvirus for which a vaccine has been developed. The VZV virulence is attenuated by passage through cell cultures, allowing the virus to develop innocuous polymorphisms, hence becoming a live-attenuated virus [3].

In a prospective study of 158 cases of herpes zoster of the head and limbs in an outpatient setting, post-herpetic neuralgia (PHN) was found in 31% of cases and segmental zoster paresis in 19% of cases. The reduction of sensory action potential amplitudes (SNAPs) and compound muscle action potentials was frequent. Sensory and motor conduction velocities were always normal, hence implying an axonal pathology. Acute denervation on electromyography was seen in 36% of cases. The number of dermatomes displaying blisters was the only variable predictive of improvement of PHN. The site of damage may be the motor roots, the plexus, or the peripheral nerve. Antiviral therapy seems to reduce the incidence of segmental zoster paresis and the severity of damage to the peripheral nerves [4].

We present the case of a 77-year-old-man who developed shingles in a cervical C8 dermatome distribution, who subsequently developed weakness of his non-dominant left hand. Careful examination and electrophysiology studies unequivocally localized the lesion to the medial cord of the brachial plexus. We believe that his presentation is more likely a radiculo-plexopathy, as the dermatomal herpetic rash extended beyond the medial antebrachial cutaneous sensory and ulnar sensory nerves, all the way up into the shoulder. We review the literature of herpetic segmental paresis, the radiologic findings on MRI, and speculate why these lesions rarely reveal contrast enhancement on MRI.

## Case presentation

We present the case of a 77-year-old right-handed man who developed a shingles rash over the ulnar half of his left hand and forearm, posterior aspect of his left upper arm, all the way proximally to behind the left shoulder. He developed a stabbing and burning pain in this distribution, with a rash that blistered and then desiccated over the course of two weeks. One week after the breakout of the rash, he noted weakness of grip of the left hand with an inability to pinch with his left hand. The weakness was restricted to the left hand and reached a nadir in two weeks. The pain required a high dose of gabapentin for relief. He also received a standard regimen of the anti-viral, famciclovir, for shingles.

His past medical history was significant for hypertension, hyperlipidemia, and chronic obstructive airway disease. His medications included lisinopril, atorvastatin, and gabapentin. He formerly smoked one pack a day and quit smoking in 1974.

On examination, his blood pressure (BP) was 120/84, pulse 76 beats per minute, height six feet, weight 202 pounds, and body-mass index (BMI) 27.4. Precordial auscultation revealed no murmurs, and carotid auscultation was negative for a bruit. His gait cadence and stability were normal. He was able to stand on his heels and toes. His speech was fluent. His cranial nerve examination was normal. Of note, there was no evidence of Horner's syndrome.

**Figure 1:**
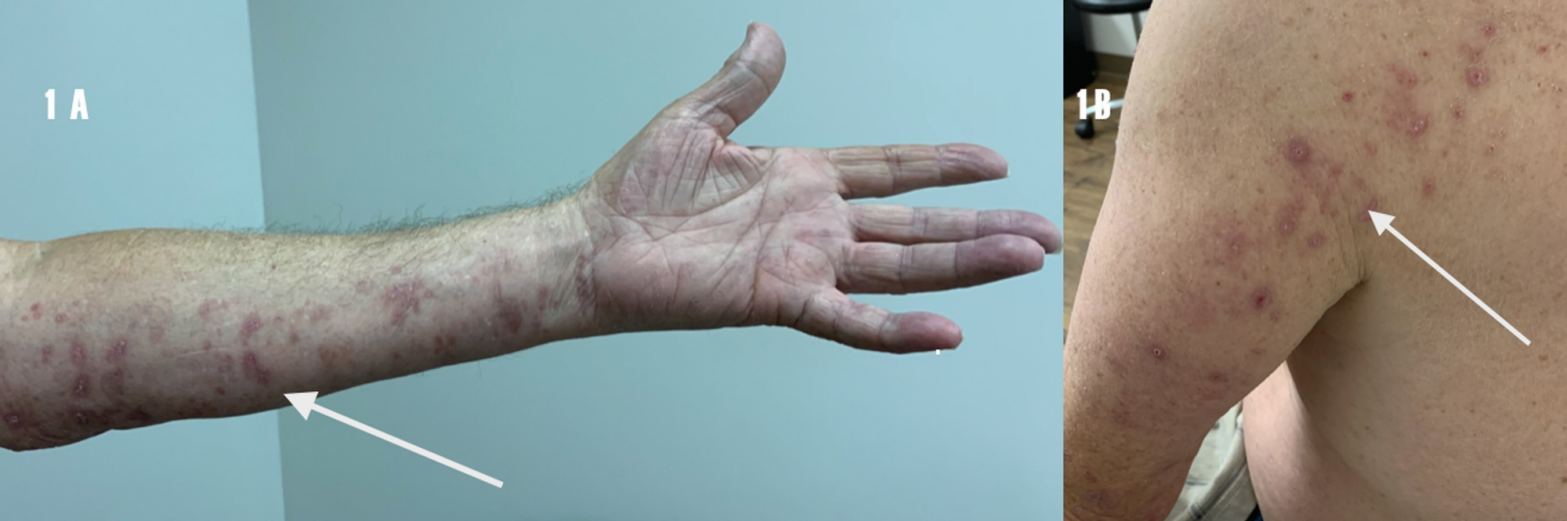
1 A. Rash of shingles over the C8 dermatome ulnar half of the left hand and forearm (white arrow). 1 B. Over the posterior aspect of the left upper arm and behind the left shoulder (white arrow) C: cervical

The power of the right upper extremity and lower extremities was graded at 5/5 by using Medical Research Council (MRC) grading. The left upper extremity grading of power revealed deltoids, biceps, coracobrachialis, brachialis, triceps, and wrist extensors and flexors at 5/5. Pronator teres was also graded at 5/5. Left flexor pollicis longus was graded at 2/5 and finger flexion at the metacarpophalangeal joints and proximal and distal interphalangeal joints at 3/5. Abductor pollicis brevis was graded at 3/5. Finger spreaders (interossei) were graded at 2/5. Mild atrophy of the left first dorsal interosseus, abductor pollicis brevis, and ulnar half of the left forearm was noted. The sensory exam revealed hyperesthesia to touch over the left C8 distribution, as outlined in Figure 1. 

**Figure 2:**
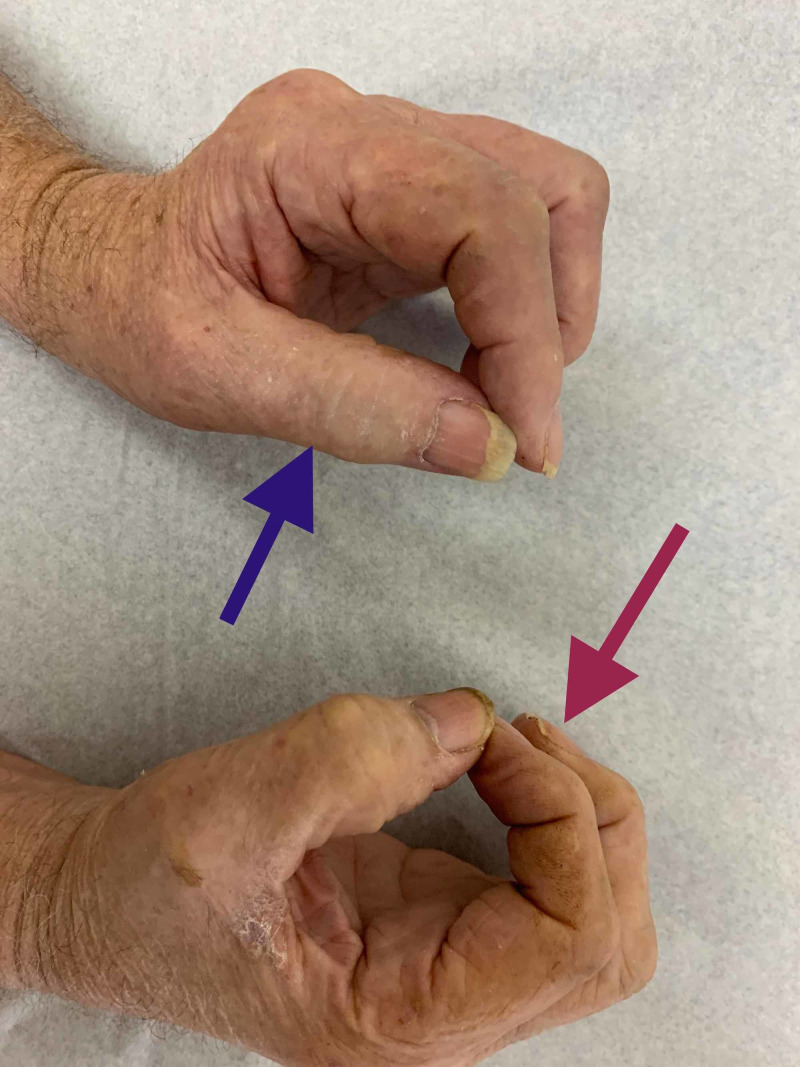
Pinch " O " sign "O" configuration with right index finger flexion and thumb flexion due to intact flexor pollicis longus and flexor digitorum superficialis muscle action (red arrow). Absent "O" configuration of the left hand due to weak inter-phalangeal thumb flexion and weak flexion of the distal interphalangeal joints of the index finger (blue arrow).

Weakness of the left flexor pollicis longus and flexor digitorum superficialis explains the pinch " O " sign, which is the inability to form the letter " O " by flexion of the thumb and flexion of the distal interphalangeal joints of the index finger during apposition of the thumb and index finger. This is demonstrated below and compared to the right hand (Figure 2).

**Figure 3:**
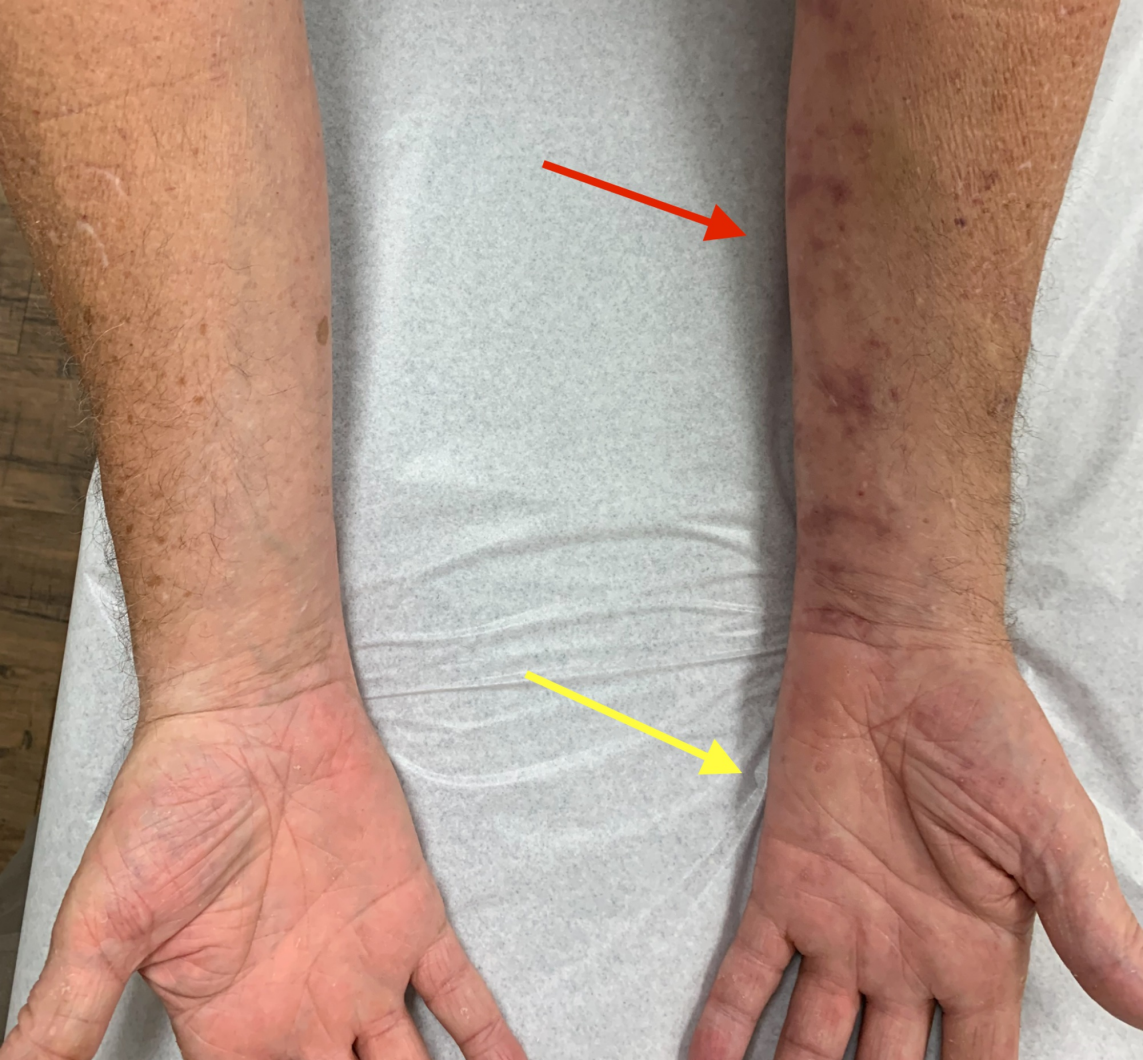
Atrophy of ulnar half of the left forearm (red arrow) and left hypothenar muscles (yellow arrow)

Atrophy of the ulnar half of the left forearm and left hypothenars reflect the pattern of weakness (Figure 3).

The next step was to confirm these findings with a nerve conduction study/electromyogram (NCS/EMG). This demonstrated a reduced left median motor amplitude, markedly reduced left ulnar motor amplitude, normal left median sensory, and diminished left ulnar sensory amplitude. The left lateral antebrachial cutaneous sensory was preserved with an absent left medial antebrachial cutaneous sensory amplitude. Left radial motor and sensory amplitudes were normal. This combination of findings of abnormal left median and ulnar nerves, with absent left medial antebrachial cutaneous sensory amplitude and preserved median sensory amplitude, is highly suggestive of a left medial cord plexopathy. The lack of radial nerve involvement precludes the lower trunk of the brachial plexus; note normal left radial motor and sensory amplitudes, also noting that the median sensory nerve emerges from the lateral and not from the medial cord of the brachial plexus (Table 1).

**Table 1:** Nerve conduction study Comparison of the left and right arm - note diminished left ulnar and median motor amplitudes, with preservation of left radial motor amplitude. Note also absent left medial antebrachial cutaneous sensory nerve and preserved left lateral antebrachial cutaneous sensory amplitude. Note normal median sensory amplitude. Abductor pollicis brevis (APB), abductor digiti minimi (ADM), extensor indicis proprius (EIP), milliVolt (mV), microVolt (micV), reduced (R)

	MOTOR AMPLITUDE mV (RIGHT ARM)	MOTOR AMPLITUDE mV (LEFT ARM)		SENSORY AMPLITUDE micV (RIGHT ARM)	SENSORY AMPLITUDE micV (LEFT ARM)
MEDIAN MOTOR (APB)	4.4	1.3 (R)	MEDIAN SENSORY	not done	10.6
ULNAR MOTOR (ADM)	5.3	0.50 (R)	ULNAR SENSORY	not done	7.0 (R)
RADIAL MOTOR (EIP)		2.3	RADIAL SENSORY	not done	13.7
			MEDIAL ANTEBRACHIAL CUTANEOUS SENSORY	not done	0 (R)
			LATERAL ANTEBRACHIAL CUTANEOUS SENSORY	not done	9.3

**Figure 4:**
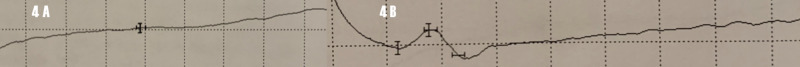
Nerve conduction study 4A: Absent left medial antebrachial cutaneous sensory amplitude. 4B: Normal left lateral antebrachial cutaneous sensory amplitude. NCS: nerve conduction study

It should be noted that the sensory and motor velocities and distal motor latencies of the left median and ulnar nerves were normal or slightly reduced and are not listed in the table. There was also no evidence of ulnar nerve entrapment at the left elbow. The reduced ulnar sensory amplitude with absent left medial antebrachial cutaneous sensory amplitude with preserved left lateral cutaneous antebrachial cutaneous and median sensory amplitudes are demonstrated graphically below (Figure 4).

The electromyography (EMG) study reveals acute and florid denervation of the left median and ulnar innervated muscles of the hand and forearm, with the preservation of radial, axillary, and musculocutaneous nerves (Table 2).

**Table 2:** EMG of left upper extremity A pattern of acute denervation of ulnar and median motor innervated muscles, with preservation of radial motor innervated muscles, suggests a medial cord plexopathy. Note the absence of lower cervical paraspinal muscle denervation. EMG grading from 1 + to 4 + as per standard fibrillation/positive wave activity when muscles are electrically studied using EMG needles. C: cervical; T: thoracic (T); EMG: electromyography

LEFT ARM MUSCLES	NERVE ROOT	SPONTANEOUS ACTIVITY	MOTOR UNIT MORPHOLOGY	iNTERFERENCE PATTERN
First dorsal interosseus	C7 C8 T1	3 +	Large PPMU	Reduced
Abductor pollicis	C8 T1	2+	Large PPMU	Reduced
Flexor digitorum profundus (median)	C8 T1	3+	Large PPMU	Reduced
Flexor digitorum profundus (ulnar)	C7 C8 T1	3+	Large PPMU	Reduced
Flexor pollicis longus	C7 C8 T1	2+	Large PPMU	Reduced
Flexor digitorum superficialis	C8 T1	2+	Large PPMU	Reduced
Extensor carpi radialis longus	C6 C7	none	Normal	Full
Biceps brachii	C5 C6	none	Normal	Full
Triceps	C6 C7 C8	none	Normal	Full
Deltoids	C5 C6	none	Normal	Full
Pronator teres	C6 C7	none	Normal	Full
Brachialis	C5 C6	none	Normal	Full
Supinator	C5 C6 C7	none	Normal	Full
Lower cervical paraspinals		None		

**Figure 5:**
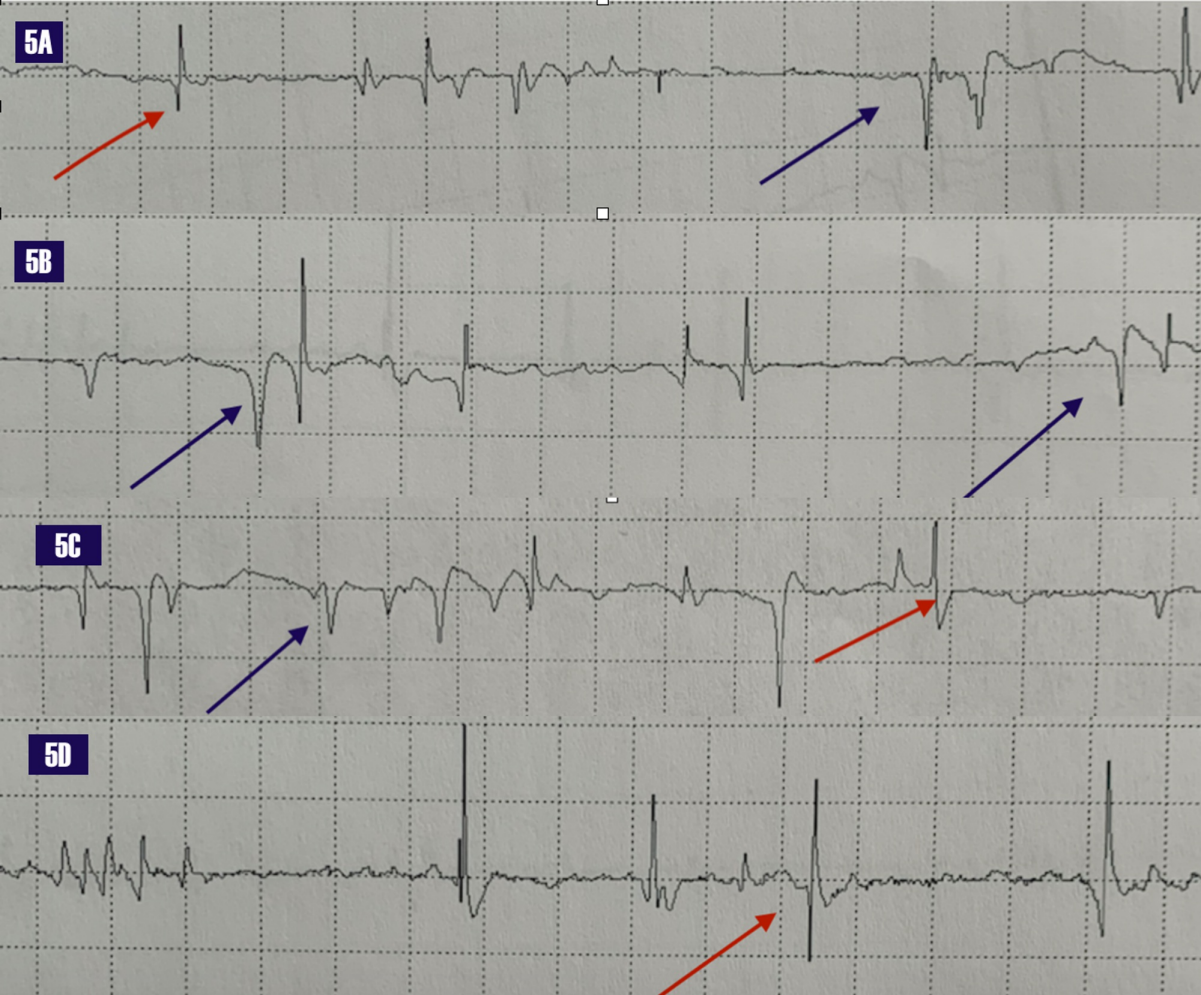
Electromyogram: Acute denervation of muscles 5A - Abductor pollicis brevis. 5B - First dorsal interosseus. 5C - Flexor pollicis longus. 5D - Flexor digitorum profundus Fibrillation potentials (red arrows), positive sharp waves (blue arrows) EMG: Electromyogram

Selected EMG of the involved muscles of the left hand and forearm reflecting the clinical pattern of weakness on examination; noting acute and florid denervation with fibrillation potentials and positive waves of involved muscles (Figure 5).

One should also emphasize the absence of left lower paraspinal muscle denervation precluding cervical C8 motor nerve root involvement. However, cervical C8 sensory nerve root involvement is not excluded, as the rash extends beyond the territory of the ulnar sensory and medial antebrachial cutaneous sensory nerves, to behind the left upper arm and left shoulder. It is more than likely that we are dealing with a C8 radiculo-medial cord brachial plexopathy. The patient was referred to physical and occupational therapy and will be monitored closely.

## Discussion

Lower trunk plexopathies are similar to cervical C8 and thoracic T1 polyradiculopathies and medial cord plexopathies. They all cause median and ulnar nerve innervated muscle weakness, mostly hand weakness. Lower trunk plexopathy is differentiated from medial cord involvement by the weakness of radial cervical C8 innervated muscles, with weakness of finger, thumb, and wrist extension. Median sensory nerve amplitude potentials (SNAPs) are preserved in both lower trunk and medial cord plexopathies as the median SNAP is a lateral cord function. The ulnar and medial antebrachial sensory amplitudes are reduced. Cervical paraspinal denervation is only seen with radiculopathy. In summary, the motor fibers of the median and ulnar nerves originate from the cervical C8 and thoracic T1 nerve roots, lower trunk, and medial cord of the brachial plexus.

Medial cord plexopathy looks like a lower trunk plexopathy, except that the radial motor nerves are preserved in a medial cord plexopathy. Only ulnar and median motor innervated muscles are involved, as in our patient. Ulnar and medial antebrachial SNAPs are reduced while the median SNAP is preserved. Median and ulnar motor amplitudes are reduced but radial motor amplitude to the extensor indicis proprius (EIP) will be preserved. The sensory fibers of the median nerve arise from the cervical C5 and C6 nerve roots, upper trunk, and the posterior cord of the brachial plexus. They are unaffected by medial cord lesions.

Upper trunk plexopathy causes weakness in the shoulder girdle muscles, mainly the deltoids and biceps. There is a reduction in median (thumb, index fingers), radial, and lateral antebrachial cutaneous SNAPs. The median (middle and ring fingers), ulnar, and medial antebrachial cutaneous sensory are normal.

A posterior cord plexopathy affects the radial, axillary, subscapular, and thoracodorsal nerves. There is a weakness of shoulder abduction, adduction, upper arm extension, and wrist and finger drop. The radial SNAP is reduced.

A middle trunk plexopathy causes weakness of elbow extension with wrist and finger drop. The median sensory to the middle finger originates in the C7 nerve root and goes through the middle trunk and thence to the lateral cord of the brachial plexus.

A lateral cord plexopathy involves the biceps, coracobrachialis, pronator trees, and flexor carpi radialis. Median SNAP to the thumb, index, and half of the ring fingers and the lateral antebrachial cutaneous SNAP are reduced. These findings are summarized in Table 3 [5].

**Table 3:** Demonstrating the different patterns of weakness of various components of the brachial plexus and sensory involvement; lateral antebrachial cutaneous (LABC) sensory nerve, medial antebrachial cutaneous (MABC) sensory nerve

	MOTOR WEAKNESS	SENSORY LOSS
UPPER TRUNK	deltoid, biceps	median (thumb, index), radial, LABC sensory nerves
POSTERIOR CORD	axillary nerve (deltoid/teres minor), radial nerve, subscapularis, and thoracodorsal nerves	radial sensory
MIDDLE TRUNK	elbow extension, wrist and finger drop	median sensory to middle finger
LATERAL CORD	biceps, coracobrachialis, pronator teres, flexor carpi radialis	median sensory to thumb, index and half of ring finger, LABC
LOWER TRUNK	median, ulnar, and radial motor	ulnar and MABC
MEDIAL CORD	median and ulnar	ulnar and MABC

**Figure 6:**
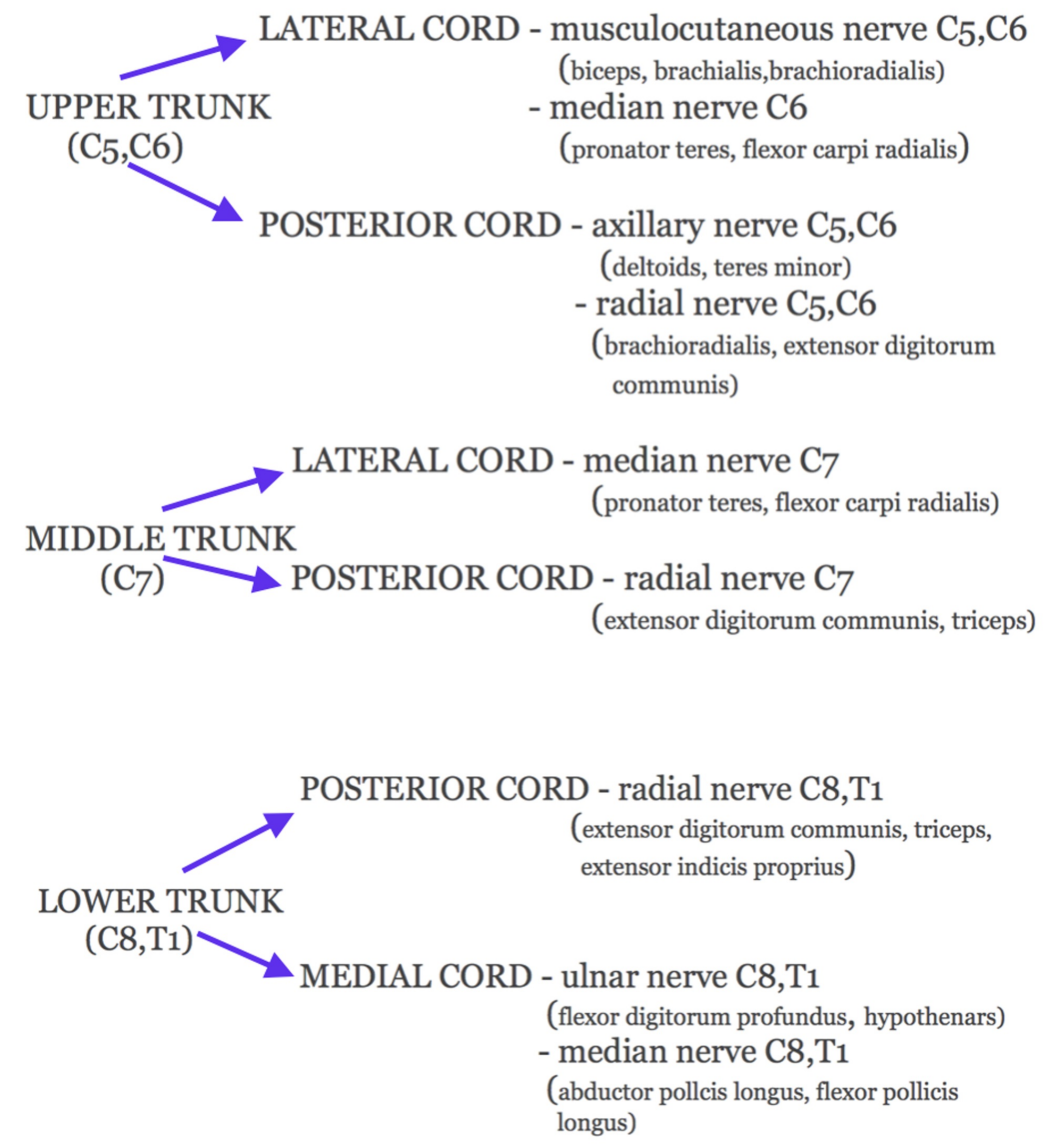
Brachial plexus: a clinically applicable branching pattern

A user-friendly pragmatic summary of the divisions of the brachial plexus is demonstrated below with deliberate omission of the anterior and posterior divisions of the trunks in order to minimize complexity and allow ease of application (Figure 6). 

We can deploy Table 3 and Figure 5 in deconstructing brachial plexopathies. For instance, in medial cord plexopathy, the main nerves and the muscles they innervate can be read from Figure 5; then, we default to Table 3 and follow the medial cord cell and note that the MABC and ulnar sensory nerves are reduced or absent, with preservation of the MABC and median sensory, as in our case. These diagrams are user-friendly and obviate the need for mnemonics or rote learning.

MRI abnormalities were found in 70% of patients with herpes zoster-associated brachial plexopathy in a study of 10 patients. T2 signal hyper-intensity of the nerves on MRI was the most frequent abnormality, found in half of the patients, followed by nerve hypertrophy in one-fifth. Half the patients demonstrated high T2 signal intensity of muscles on MRI, indicating denervation, especially in shoulder girdle muscles. Gadolinium enhancement was not seen despite early imaging [6].

Kawajiri S et al. reported three patients with herpes zoster-associated plexopathy. Two showed a motor weakness of the left shoulder girdle with a herpetic rash in the cervical C5 and C6 dermatomes. The third patient developed thigh weakness and a herpetic rash in the lumbar L2 and L3 dermatome. The electromyograms of all three patients showed acute denervation in the affected muscles. The patients were treated with intravenous acyclovir and corticosteroid pulse therapy. The patients improved [7].

Reda H et al. identified nine patients with zoster-associated neuritis; two with ulnar neuropathy, three with median neuropathy, one with femoral neuropathy, and two with sciatic neuropathy. Most patients had significant weakness in the distribution of the afflicted nerve and the weakness was associated with post-herpetic neuralgia. There was an MRI abnormality in most patients, either nerve hypertrophy or T2 signal hyperintensity, and, rarely, contrast enhancement (only one patient). The mean duration of weakness lasted 282 days [8].

Why post-herpetic brachial plexopathies or neuritides rarely present with contrast enhancement on MRI is intriguing. We postulate that early default treatment with anti-virals limits the inflammatory response, and this may potentially reduce the breakdown of the blood-nerve barrier precluding access to contrast [9]. Anti-virals such as famciclovir may have a neuroprotective effect, as prophylactic famciclovir may significantly reduce the percentage of patients experiencing postoperative delayed facial paresis following acoustic tumor resection [10]. Lastly, it is well-known that patients on famciclovir have a two-fold faster resolution of postherpetic neuralgia than placebo patients [11].

## Conclusions

We unequivocally demonstrate the rare presentation of medial cord plexopathy with segmental paresis, namely, hand weakness, following a herpes virus infection in a cervical C8 dermatome. In this article, we go further by proposing a practical method of localizing a brachial plexus lesion by deploying a diagram and a table, obviating the need for rote learning. This is an ancillary aid to a detailed examination that includes pattern recognition and deductive reasoning. Finally, we speculate on the dramatic finding that despite post-herpetic neuritis being an inflammatory disease, enhancement on MRI is peculiarly a rare finding.
